# Upper‐Limb Paresis Severity Is Associated With Practice Dosage During Task‐Oriented Training in Subacute Stroke—An Exploratory Secondary Analysis

**DOI:** 10.1002/pri.70316

**Published:** 2026-08-13

**Authors:** Subramanian Durairaj, Akhila Jagadish, John M. Solomon, Mindy F. Levin

**Affiliations:** ^1^ Department of Physiotherapy Manipal College of Health Professions Manipal Academy of Higher Education Manipal India; ^2^ Faculty of Medicine and Health Sciences School of Physical and Occupational Therapy, McGill University Montreal Quebec Canada; ^3^ Center for Interdisciplinary Research in Rehabilitation (CRIR) Montreal Quebec Canada

**Keywords:** high‐dose rehabilitation, practice dosage, stroke, task‐oriented training, upper limb

## Abstract

**Background and Purpose:**

Intensive, high‐repetition practice is one of the strategies incorporated into upper‐limb task‐oriented training (UL‐TOT) to promote experience‐dependent neural plasticity after stroke. Although a target of 300 repetitions during a one‐hour UL‐TOT session is feasible, considerable variability in the practice dosage achieved across individuals has been observed. Multiple factors may influence practice dosage during UL‐TOT. Therefore, this exploratory secondary analysis aimed to examine factors associated with practice dosage during UL‐TOT in individuals with stroke.

**Methods:**

This secondary analysis included data from 33 individuals with subacute stroke enrolled in a randomized controlled trial. Associations between age, sex, stroke type, lesion location and volume, poststroke duration, upper‐limb paresis severity, spasticity severity, and depression severity (independent variables), and practice dosage (dependent variable) were analyzed using stepwise multiple linear regression.

**Results:**

Among the variables examined, upper‐limb paresis severity was the only factor retained in the final stepwise multiple linear regression model and was significantly associated with practice dosage within this dataset (*β* = 0.804; *p* < 0.001). The regression model was statistically significant and explained 63.4% of the variance in practice dosage (adjusted *R* ^2^ = 0.634).

**Discussion:**

Upper‐limb paresis severity was the only factor significantly associated with practice dosage during therapist‐guided UL‐TOT in individuals with subacute stroke within this dataset. However, these findings should be interpreted with caution, given the small sample size and the exploratory nature of the analysis.

## 1 Introduction

Approximately 50%–80% of individuals with stroke experience upper‐limb hemiparesis during the acute phase, and nearly 50% continue to have persistent paresis in the chronic phase (Nakayama et al. 1994; Lawrence et al. 2001; Persson et al. 2012). Persistent upper‐limb paresis limits activities of daily living and reduces quality of life. Therefore, improving upper‐limb function remains a major goal of stroke rehabilitation. Recovery of upper‐limb function after stroke is influenced by the intensity and amount of task‐specific practice received during rehabilitation. Experimental and clinical evidence suggests that increasing the intensity and repetition of task‐specific practice promotes experience‐dependent neural plasticity and motor recovery, making practice dosage a key component of stroke rehabilitation (Kleim and Jones 2008; Veerbeek et al. 2014). Accordingly, task‐oriented training (TOT), which involves repetitive practice of functional tasks, is widely recommended to improve upper‐limb function after stroke (Pollock et al. 2014; French et al. 2016).

Upper‐limb task‐oriented training (UL‐TOT) builds on this principle by emphasizing intensive, high‐repetition practice to promote motor recovery. Previous studies have demonstrated that high‐repetition task‐oriented upper‐limb training is feasible in individuals with stroke, with approximately 300 repetitions performed within a one‐hour training session (Birkenmeier et al. 2010; Waddell et al. 2014; Lang et al. 2015). However, despite the feasibility of delivering high‐dose task‐oriented training, the practice dosage achieved varies considerably among individuals, posing a challenge to the delivery of high‐dose rehabilitation. Relatively little is known about the factors contributing to this variability during UL‐TOT.

In the parent randomized controlled trial (RCT), participants with subacute stroke received Trunk Restraint Task‐Oriented Training (TR‐TOT). This therapeutic approach reduces compensatory anterior trunk displacement and facilitates effective use of the paretic upper‐limb during reaching task practice. Although the intervention targeted 300 repetitions within a one‐hour session, considerable variability in the practice dosage was observed across participants. Multiple factors may influence practice dosage during UL‐TOT, including participant‐related demographic and clinical characteristics such as age, sex, stroke type, lesion location and volume, poststroke duration, upper‐limb paresis severity, spasticity severity, and depression severity. Other participant‐, task‐, and contextual factors may also contribute to variability in practice dosage. However, the present exploratory secondary analysis was limited to the demographic and clinical variables available within the parent trial dataset.

Therefore, this exploratory secondary analysis of data from an RCT aimed to identify demographic and clinical factors associated with practice dosage during high‐dose UL‐TOT in individuals with subacute stroke. Given the exploratory nature of the study, no a priori hypotheses were formulated regarding the association between these variables and practice dosage during UL‐TOT.

## 2 Methods

This study is an exploratory secondary analysis of data from an RCT. Thirty‐three community‐dwelling individuals with subacute stroke were enrolled in the parent RCT (IEC no: 735/2017; CTRI no: CTRI/2018/04/013178), which examined the effects of transcranial direct current stimulation (tDCS) on upper‐limb movement. As this study was an exploratory secondary analysis of data from a parent trial, an a priori sample size calculation specific to the regression analysis was not performed.

Participants were included and excluded based on the following criteria: Inclusion criteria: (a) Haemodynamically stable individuals with a cortical, subcortical or mixed (cortical and subcortical) brain lesion; (b) Early (14 days–3 months) or late subacute (3–6 months) phase poststroke (Bernhardt et al. 2017); (c) Ischemic or haemorrhagic stroke in the area supplied by the middle cerebral artery, anterior cerebral artery or posterior cerebral artery, confirmed by MRI/CT; (d) Both sexes and aged between 30 and 75 years; (e) Upper‐limb paresis with Chedoke McMaster Arm Score (CMSA) of 2–6 out of 7 and Hand score of 2–6 out of 7. Exclusion criteria: (a) Major neurological (other than stroke)/neuromuscular/orthopedic conditions; (b) Major cognitive deficits (Montreal Cognitive Assessment score < 23); (c) History of psychiatric disorders, seizures, migraines within the past 6 months; (d) Presence of Metal implants in the cranium or other implanted devices (e.g., cochlear, cardiac implants); (e) Current use of medications that could affect brain activity (e.g., antiepileptic and psychoactive drugs).

Participants were randomly assigned to either Group A (experimental) or Group B (control). Group A received anodal tDCS at 2 mA, with the anodal electrode (active) placed over the lesioned primary motor cortex, and the cathodal electrode over the contralateral supraorbital region. Group B received sham tDCS using the same electrode montage; 2 mA was delivered for 1 minute at the beginning and end of the session, with no current applied for the remaining 28 minutes. Both groups received TR‐TOT in combination with tDCS. TR‐TOT was delivered for 60 minutes, with tDCS applied during the first 30 minutes. The intervention was administered once daily, 5 days per week, for 4 weeks. All sessions were delivered in participants' homes by the same neurorehabilitation therapist with 17 years of clinical experience.

### 2.1 TR‐TOT Intervention

Participants were instructed to sit at the table with the affected arm positioned alongside and parallel to the trunk. The trunk was restrained to the chair backrest using two straps placed across the chest. Practice tasks were selected based on prehension type: cylindrical (simulated drinking task), spherical (ball‐into‐mug task), and hook grasp (simulated bathing task). Participants practiced three standardized functional reach‐to‐grasp tasks: (a) reach, grasp, and bring a plastic bottle to the mouth (cylindrical grasp); (b) reach, grasp, and drop a ball into a mug (spherical grasp); (c) reach, grasp, and lift a mug over the head (hook grasp).

Task practice was tailored to each participant's skill level. Tasks were designed to be challenging, and task difficulty was progressed by increasing the distance of the target from the body (from near to far), increasing the height of the target (from below shoulder level to above the shoulder level), and increasing the weight (from light to heavy) and size (from small to large) of the objects. Task difficulty was progressed when performance stabilized, defined as a standard deviation of ≤ 10 repetitions across three consecutive sessions. Manual assistance was provided when participants were unable to open their fingers for grasp‐and‐release.

In each session, each task was practiced for 20 minutes. During practice, the physical therapist manually counted the repetitions for each task and recorded the count on a standardized data collection sheet immediately after completion of each task to ensure accurate documentation. As the same therapist delivered all intervention sessions, consistency in task administration and repetition counting was intended to be maintained across participants and sessions. However, observer bias cannot be completely excluded. Active training and rest periods were also recorded. Practice intensity during each session was measured using the modified Borg Rate of Perceived Exertion scale (RPE) (Borg 1998). The practice conditions related to motor learning principles applied during UL‐TOT are presented in Table S1.

Demographic and clinical variables, including age, sex, stroke type, lesion location and volume, stroke duration, upper‐limb paresis severity, spasticity severity, and depression severity, were collected during screening and baseline assessment. Based on their availability in the parent trial dataset and their potential clinical relevance to practice dosage during UL‐TOT, these variables were selected for inclusion as independent variables in the regression analysis. These variables were considered because demographic and clinical characteristics may influence an individual's capacity to engage in repetitive task practice after stroke. The dependent variable was practice dosage, defined as the mean number of repetitions performed per participant across the 20 training sessions.

### 2.2 Image Acquisition and Data Processing for Lesion Volume Analysis

All participants were scanned on a 3 T United Imaging uMR780 MRI scanner (United Imaging Healthcare, Shanghai, China) equipped with a 32‐channel head coil. The routine clinical stroke MRI protocol included axial T1‐weighted turbo spin‐echo, fluid‐attenuated inversion recovery (FLAIR), T2‐weighted fast spin‐echo (FSE), susceptibility‐weighted imaging (SWI), and time‐of‐flight (TOF) vascular imaging.

Diffusion‐weighted imaging (DWI), which is part of standard clinical care at our institution, was acquired with three b‐values (0, 50, and 1000 s/mm^2^) along 30 diffusion encoding directions using a vendor‐supplied single‐shot, twice‐refocused spin‐echo echo‐planar imaging sequence. The number of excitations (NEX) was set as follows: *b*0 = 1, b50 = 2, and b1000 = 4. Other imaging parameters were as follows: slice thickness = 5 mm (30% gap), number of slices = 25, TR/TE = 4164/74.8 ms, field‐of‐view (FOV) = 230 × 230 mm^2^, acquisition matrix = 128 × 100, image resolution = 1.8 × 1.8 mm^2^, Bandwidth per pixel = 1800 Hz, acceleration factor = 2, and acquisition time was approximately 3.33 minutes. Apparent diffusion coefficient (ADC) maps corresponding to these images were automatically generated on the workstation using monoexponential fitting.

### 2.3 Data Processing

The diffusion‐weighted images were exported into a dedicated workstation (64‐bit PC, running Windows 10 Professional) and analyzed using the in‐house U‐Viewer software for DICOM visualization and annotation. Lesion volume was estimated using the ABC/2 method, where A represents the maximum transverse diameter (in cm) and B represents the maximum longitudinal diameter (in cm), both measured on the axial DWI slice showing the largest lesion extent. C represents the number of slices on which the lesion is visible multiplied by the slice thickness (in cm). All analyses were conducted by a trained research scholar with 8 years of experience in medical imaging technology.

Lesion volume data were unavailable for nine participants because neuroimaging data could not be retrieved from hospital records. Some participants were recruited directly from the community and had received treatment at other hospitals; therefore, their imaging data were unavailable. Missing lesion volume values were imputed using multiple imputations. Fifty imputed datasets were generated to account for the uncertainty associated with missing data, and stepwise linear regression analyses were subsequently performed on the imputed datasets.

Upper‐limb paresis severity was assessed using the Shoulder Abduction and Finger Extension (SAFE) score. The SAFE score is widely used to quantify upper‐limb motor weakness following stroke (Stinear et al. 2012, 2017). It is calculated as the sum of the Medical Research Council (MRC) muscle strength grades (0–5) for shoulder abduction and finger extension, with a maximum score of 10. Based on the SAFE score, upper‐limb weakness is classified as severe (0–4), mild or moderate (5–8), or little to no weakness (> 8) (Simpson et al. 2021). Higher SAFE scores indicate greater muscle strength in the paretic upper‐limb.

The severity of elbow flexor spasticity was assessed using the Composite Spasticity Index (CSI), a clinical measure comprising three components: tendon jerk (score: 0–4 points), resistance to passive stretch (score: 0–8 points), and clonus (score: 0–4 points) with a maximum score of 16. The CSI score was classified as mild (0–9), moderate (10–12), and severe (13–16) spasticity (Chan 1986; Calota and Levin 2009). Higher CSI scores indicate greater spasticity.

The severity of depression was evaluated using the Patient Health Questionnaire (PHQ‐9), a self‐report measure consisting of nine items. Each item was scored on a 4‐point scale (0–3), with a maximum score of 27. PHQ‐9 scores are categorized as none or minimal (0–4), mild (5–9), moderate (10–14), moderately severe (15–19), and severe (20–27) depression (Kroenke et al. 2001; Kroenke and Spitzer 2002).

### 2.4 Statistical Analysis

Missing lesion volume data were handled using multiple imputation with the Fully Conditional Specification (FCS) method before statistical analyses. Fifty imputed datasets were generated. Continuous descriptive statistics were calculated from the multiply imputed datasets, whereas categorical variables were summarized using the observed data. Before the regression analysis, all continuous variables were assessed for normality using the Shapiro‒Wilk test. Normally distributed continuous variables were summarized as means and standard deviations; non‐normally distributed continuous variables as medians and interquartile ranges; and categorical variables as frequencies and percentages.

The associations between baseline demographic and clinical variables, tDCS group (independent variables), and practice dosage (dependent variable) were examined using stepwise multiple linear regression on the 50 imputed datasets. A stepwise approach was selected because of the exploratory nature of the study and its aim to identify variables associated with practice dosage during UL‐TOT rather than to test a predefined theoretical model. Given the relatively small sample size in relation to the number of variables examined, the analysis was considered exploratory. The significance levels for variable entry and removal were set at *p* ≤ 0.05 and *p* ≥ 0.10, respectively.

Assumption testing was conducted as part of the planned regression analysis. Diagnostic checks included linearity and homoscedasticity (evaluated using residual scatter plots), normality of residuals (assessed using a Q‒Q plot), and multicollinearity (assessed using variance inflation factors [VIFs] and tolerance values). Regression coefficients were initially examined, and diagnostic checks were performed to assess model assumptions. The final interpretation of the model was based on the confirmation that the assumptions were adequately met. Model performance was evaluated using the coefficient of determination (*R* ^2^), adjusted *R* ^2^, and the standard error of the estimate (SEE). All analyses were performed using IBM SPSS Statistics for Windows (version 30.0.0).

## 3 Results

A total of 33 participants were included in the study. The mean (SD) age was 52.3 (12.9) years. Regarding poststroke duration, 22 (66.7%) participants were in the early subacute phase, and 11 (33.3%) were in the late subacute phase. Based on SAFE scores, 22 (66.7%) participants had a severe upper‐limb paresis (SAFE score ≤ 4), while 11 (33.3%) had mild to moderate paresis (SAFE score 5–8). Regarding spasticity severity, 30 (90.9%) participants had mild elbow flexor spasticity, and 3 (9.1%) had moderate spasticity. In terms of depression severity, 10 (30.3%) participants had none or minimal depression, 12 (36.5%) had mild depression, 7 (21.1%) had moderate depression, and 4 (12.1%) had moderately severe depression.

Lesion volume data were unavailable for nine participants (27.3%) and were handled using multiple imputation. The median lesion volume was 21.8 cm^3^ (Q1–Q3: 6.9–36.6 cm^3^). Regarding lesion location, 3 (9.1%) participants had cortical lesions, 23 (69.7%) had subcortical lesions, and 7 (21.2%) had mixed cortical and subcortical lesions. Participant characteristics and descriptive statistics of variables included in the regression analysis are presented in Table 1. Detailed demographic and clinical characteristics of the participants are presented in Table S2, and the practice dosage achieved during UL‐TOT across participants is presented in Table S3. The mean (SD) practice dosage was 137 (89) repetitions per session, indicating substantial inter‐individual variability.

**TABLE 1:** Participant characteristics and descriptive statistics of variables included in the regression analysis (*N* = 33).

Variables (independent variables)	Values
*N*	33
Age (years), mean ± SD	52.3 ± 12.9
Sex (male/female), *n* (%)	23 (69.7)/10 (30.3)
Type of stroke (ischemic/hemorrhagic/both), *n* (%)	19 (57.6)/11 (33.3)/3 (9.1)
Lesion location (cortical/subcortical/mixed), *n* (%)	3 (9.1)/23 (69.7)/7 (21.2)
Lesion volume (cm^3^), median (Q1–Q3)	21.8 (6.9–36.6)
Time since stroke (days), mean ± SD	76.1 ± 49.4
SAFE score, median (Q1–Q3)	2.0 (0.0–5.0)
CSI score of elbow flexors, mean ± SD	7.1 ± 2.1
PHQ‐9 score, mean ± SD	7.6 ± 4.8
Anodal tDCS/Sham tDCS, *n* (%)	17 (51.5)/16 (48.5)

*Note:* Data are presented as mean ± SD, median (Q1–Q3), or *n* (%), as appropriate. Continuous descriptive statistics are based on the multiply imputed dataset (50 imputations), whereas categorical variables are based on the observed data.

Abbreviations: Both = ischaemic and hemorrhagic; CSI = composite spasticity index; Mixed = cortical and subcortical; PHQ‐9 = patient health questionnaire‐9; Q1 = first quartile; Q3 = third quartile; SAFE = shoulder abduction and finger extension; SD = standard deviation.

In the stepwise multiple linear regression analysis, upper‐limb paresis severity (SAFE score) was the only variable retained in the final model and was significantly associated with practice dosage during UL‐TOT within this dataset (*β* = 0.804; *p* < 0.001). Age, sex, stroke type, stroke duration, lesion location, lesion volume, spasticity severity, depression severity, and tDCS group were not retained in the final model. The regression model was statistically significant [*F*(1, 31) = 56.499, *p* < 0.001] and explained 63.4% of the variance in practice dosage (adjusted *R* ^2^ = 0.634, *R* ^2^ = 0.646).

Assessment of regression assumptions using diagnostic plots and collinearity statistics indicated no violations of linearity, homoscedasticity, normality of residuals, or multicollinearity. The scatter plot of standardized residuals versus standardized predicted values (Figure S1) and the normal Q–Q plot of standardized residuals (Figure S2) are provided in the supplementary material. The results of the final stepwise multiple linear regression model are presented in Table 2.

**TABLE 2:** Final stepwise multiple linear regression model examining variables associated with practice dosage during UL‐TOT (*N* = 33).

Variable	Unstandardized B	Standard error	Standardized *β*	*t*	*p*‐value	95% CI for B	Tolerance	VIF
Constant	39.277	16.090	—	2.441	0.015	7.740 to 70.813	—	—
SAFE	30.856	4.105	0.804	7.517	< 0.001	22.810 to 38.902	1.000	1.000

*Note: R* ^2^ = 0.646; Adjusted *R* ^2^ = 0.634; *F*(1, 31) = 56.499; SEE = 53.979; *p* < 0.001.

Abbreviations: CI = confidence interval; *R* ^2^ = coefficient of determination; SAFE = shoulder abduction and finger extension; SEE = standard error of estimate; UL‐TOT = upper‐limb task‐oriented training; VIF = variance inflation factor.

## 4 Discussion

This exploratory secondary analysis examined factors associated with practice dosage during therapist‐guided UL‐TOT in individuals with subacute stroke. Among the prespecified factors examined, upper‐limb paresis severity (SAFE score) was the only factor retained in the final stepwise multiple linear regression model and was significantly associated with variation in practice dosage.

Participants with severe upper‐limb paresis had a mean (SD) practice dosage of 95 (66) repetitions per session, whereas those with mild to moderate paresis had a mean (SD) of 222 (68) repetitions per session. One possible explanation is that individuals with more severe paresis often have longer movement times, lower peak velocities, and more segmented movement patterns during reach‐to‐grasp tasks than those with mild paresis (Murphy et al. 2010; Michaelsen et al. 2004). However, considerable variability in practice dosage was observed within both groups. While most participants with severe paresis achieved a lower practice dosage (fewer than 100 repetitions per session), some achieved substantially higher dosages. Similarly, although participants with mild to moderate paresis generally achieved higher practice dosage, substantial variability remained evident within this group. These findings suggest that practice dosage during UL‐TOT may be partly constrained by paresis severity; however, the substantial variability observed in both groups indicates that other unmeasured factors likely contributed to it.

In contrast to upper‐limb paresis severity, the other prespecified factors, including age, sex, poststroke duration, stroke type, lesion location, lesion volume, spasticity severity, depressive symptoms, and tDCS allocation, were not retained in the final stepwise regression model.

The lack of observed associations between these other prespecified participant characteristics and practice dosage should be interpreted with caution, as the study sample was unevenly distributed across several participant characteristics, including age, sex, poststroke duration, stroke type, lesion location, and spasticity severity. In addition, lesion volume data were unavailable for nine participants and were handled using multiple imputation, while individuals with severe spasticity were excluded from the parent trial. Collectively, these factors, together with the small sample size and substantial inter‐individual variability in practice dosage, may have limited the ability to detect independent associations between the other prespecified factors and practice dosage. Although upper‐limb severity was also unevenly distributed within the study sample, with 67% of participants having severe paresis and 33% having mild‐to‐moderate paresis, it remained the only prespecified factor associated with practice dosage.

Although it is clinically expected that individuals with greater upper‐limb paresis would achieve fewer repetitions during UL‐TOT, the novelty of this study lies in quantifying this association within a therapist‐guided high‐dose training program. Previous studies have primarily focused on the feasibility of delivering high‐repetition TOT or on predictors of functional recovery after stroke. In contrast, the present exploratory analysis examined practice dosage as the outcome measure and assessed the associations between multiple demographic and clinical factors and the achieved practice dosage. These findings extend previous work by providing quantitative evidence that upper‐limb paresis severity was the only prespecified factor independently associated with practice dosage in this dataset. However, substantial variability in practice dosage was observed even among individuals with similar levels of paresis severity, suggesting that factors beyond paresis severity also contribute to this variation. These findings are preliminary and hypothesis‐generating. Further research with larger, more diverse samples and the inclusion of additional potentially relevant factors is needed to confirm these findings and improve our understanding of factors contributing to variability in practice dosage among individuals with upper‐limb paresis.

Overall, this exploratory analysis suggests that upper‐limb paresis severity may be associated with variation in practice dosage during therapist‐guided UL‐TOT in individuals with subacute stroke. There was insufficient evidence in this sample to support independent associations between the other demographic and clinical variables examined and practice dosage during UL‐TOT. Given the relatively small sample size, the uneven distribution of participants across several factors, and missing lesion volume data, the absence of statistically significant associations should not be interpreted as evidence that these factors are unrelated to practice dosage. Several potentially important determinants of practice dosage, including fatigue, pain, motivation, day‐to‐day mood, attention, self‐efficacy, other task‐related factors, and contextual factors, were not available in the present dataset and therefore could not be evaluated. Consequently, the present model should not be interpreted as a comprehensive explanation of variability in practice dosage.

Practice dosage, quantified as the number of repetitions achieved during training, represents a process measure of therapy exposure rather than a direct patient‐centered outcome such as functional recovery, participation, or quality of life. However, it reflects the amount of task‐specific practice completed during UL‐TOT and provides valuable information about rehabilitation delivery. Recent evidence and stroke rehabilitation guidelines emphasize the importance of delivering intensive, task‐specific upper‐limb rehabilitation while optimizing therapy dosage in clinical practice (Donnellan‐Fernandez et al. 2022; Murphy et al. 2025). Accordingly, the present findings should be interpreted as describing variability in achieved practice dosage during training rather than the effectiveness of the intervention. The observed association between upper‐limb paresis severity and practice dosage provides a rationale for future studies investigating strategies tailored to different levels of impairment to facilitate achieving higher practice dosages during high‐dose task‐oriented rehabilitation. However, given the exploratory nature of the present study, these implications should be interpreted cautiously and require confirmation in larger prospective studies.

This study has several limitations. The sample size was relatively small (*N* = 33), reducing statistical power and limiting the stability of the regression estimates. Although missing lesion volume data were handled using multiple imputation, the relatively high proportion of missing values (27.3%) introduced uncertainty into the regression estimates. As this was a secondary analysis of data from a parent trial, the available factors were limited to those originally collected. Therefore, several potentially relevant participant‐, task‐, and contextual factors could not be examined. The findings are not generalizable to individuals in the acute or chronic stages of stroke or to those with severe spasticity, who were not represented in this sample. Furthermore, the uneven distribution of several baseline characteristics, including age, sex, stroke type, lesion location, poststroke duration, and spasticity severity, limited the ability to detect associations and reduced the generalizability of the findings. Although the regression model explained a substantial proportion of the variance in practice dosage (*R* ^2^ = 0.646), this estimate should be interpreted cautiously. Given the relatively small sample size, evaluation of multiple candidate variables, and use of stepwise regression in this exploratory study, the model may be unstable and prone to overfitting. Accordingly, the reported *R* ^2^ may represent an optimistic estimate of model performance and may not generalize to other samples.

### 4.1 Implications of Physiotherapy Practice

The findings suggest that individuals with more severe upper‐limb paresis achieved lower practice dosage during UL‐TOT than those with less severe paresis. However, considerable variability in practice dosage was observed within both paresis severity groups, suggesting that factors beyond paresis severity also contributed to this variability. These findings highlight the importance of recognizing inter‐individual variability in practice dosage when planning and monitoring high‐dose upper‐limb task‐oriented rehabilitation. Further research is needed to identify additional factors associated with variability in practice dosage.

## Author Contributions

Subramanian Durairaj contributed to the conception and study design, data analysis, writing, drafting, editing, and manuscript revision and submission. Akhila Jagadish contributed to data collection, editing, and manuscript revision. John M. Solomon contributed to the conception and design of the study, reviewed the manuscript, and provided final approval for publication. Mindy F. Levin contributed to the conception and design of the study, reviewed the manuscript, and provided final approval for publication. All the authors have read and approved the final version of the manuscript.

## Funding

The authors have nothing to report.

## Ethics Statement

The original randomized controlled trial was approved by the Institutional Ethics Committee of Kasturba Medical College and Kasturba Hospital (IEC No. 735/2017) on 15 November 2017 and was registered with the Clinical Trials Registry‐India (CTRI No. CTRI/2018/04/013178) on 11 April 2018. For this secondary analysis, only de‐identified data from the parent trial were used, and no new data were collected from participants. Therefore, additional ethics approval was not required.

## Consent

Informed consent was obtained from all participants involved in the parent randomized controlled trial.

## Conflicts of Interest

The authors declare no conflicts of interest.

## Supporting information


**FIGURE S1:** Scatter plot of standardized residuals versus standardized predicted values for the final stepwise multiple linear regression model of practice dosage during UL‐TOT.

**FIGURE S2:** Normal Q–Q plot of standardized residuals from the final stepwise multiple linear regression model of practice dosage during UL‐TOT

**TABLE S1:** Practice conditions related to motor learning principles applied during UL‐TOT

**TABLE S2:** Demographics and clinical characteristics of participants (*N* = 33).

**TABLE S3:** Practice dosage achieved during UL‐TOT across participants (*N* = 33).

## Data Availability

The data that supports the findings of this study are available in the supplementary material of this article.
