# Supplementary material for: Upper‐Limb Paresis Severity Is Associated With Practice Dosage During Task‐Oriented Training in Subacute Stroke—An Exploratory Secondary Analysis
Source: Physiother Res Int. 2026 Aug 13;31(4):e70316. doi: 10.1002/pri.70316 (PMC13470900; doi:10.1002/pri.70316)
Supplement: Supplementary file 1 [file PRI-31-e70316-s001.docx]

**Table S1: Practice conditions related to motor learning principles applied during UL-TOT.**

| **Practice conditions** | **Description** |
| --- | --- |
| Practice distribution | Massed practice: Individuals practiced actively for 1 hour, with rest provided only after completing each task (rest only after 20 minutes of training). The rest period ranges from 2 to 3 minutes. A rest period was also given at the individual’s request. |
| Practice variability | Variable practice: Three functional reach-to-grasp tasks were practiced in a sequential order with the simulated drinking task, followed by the ball-into-mug task, and then the simulated bathing task. Within each task, object characteristics such as height, distance, weight, and size were varied to introduce variability in practice (within-task variability). |
| Practice order | Random order: Participants reached for targets arranged in random order. |
| Movement complexity | The whole task practice was provided. |
| Challenging and progressive task practice | The following methods provided challenging and progressive task practice:  Distance and height of the targets:  The distance and height of the target positions for the reaching task were customized for each participant by determining each person’s maximal voluntary elbow extension and shoulder flexion for reaching various areas on the table. Then the target position was set slightly beyond their maximum reach on the table.  The distance and height of the target position were determined as follows: The workspace on the table was divided vertically into two zones: zone 1 (below shoulder level) and zone 2 (above shoulder level). In both zones 1 and 2, the workspace was again divided horizontally into four zones: zone 1 (ipsilateral near target), zone 2 (contralateral near target), zone 3 (ipsilateral far target), and zone 4 (contralateral far target). Initially, subjects were encouraged to reach for the horizontal targets in zone 1 (below shoulder level), starting from the ipsilateral near target, progressing to the contralateral near target, then to the ipsilateral far target, and to the contralateral far target, then progressing to the horizontal targets in zone 2 (above shoulder level). The height of the target was adjusted using stools of different sizes.  Weight and size of the object:  The weight and size of the object were challenged and progressed by starting with small weights (e.g., less water in the water bottle) and a small size based on the individual’s strength and hand opening. The weight of the plastic bottle ranged from 15 g to 35g (15g, 20g, 25g, 35g) when empty and from 205g to 1800g (205g, 280g, 295g, 540g, 715g, 810g, 1.030g, and 1800g) when full. The bottle circumferences ranged from 15.1 cm to 28 cm (15.1 cm, 16.8 cm, 18 cm, 18.2, 20 cm, 22 cm, 24 cm, 27 cm, 28 cm). The weight of the ball ranged from 5g to 35g (5g, 15g, 20g, 30g, 35g, 75g, and 165g), and the circumference of the ball ranged from 8 cm to 25 cm (8 cm, 10 cm, 11.3 cm, 12.2, 14 cm, 21.5 cm, 22.2 cm, 25 cm). Two mugs with a circumference of 35 cm and 45 cm were also used. |
| Dosage | Amount of practice: quantified as both duration and number of repetitions.  Duration: Participants completed 1 hour of active training, excluding rest periods, with each task practiced for 20 minutes.  Number of repetitions:   A) Simulated drinking task: One repetition was defined as the participant lifting their arm from the starting position, reaching for the plastic bottle, grasping and bringing it to their mouth, returning the bottle to the target position, and then returning the arm to its initial position. B) Ball-into-mug task: Each repetition involved the participant lifting their arm from the starting position, reaching for the ball, grasping and lifting it, releasing the ball into a mug, and returning the arm to its initial position. C) Simulated bathing task: One repetition consisted of the participant lifting their arm from the starting position, reaching for the mug, grasping it, lifting the mug overhead, replacing the mug in the target position, and returning the arm to its initial position.   Frequency: 1 session per day, 5 sessions per week  Total duration of training: 4 weeks. |
| Type of feedback | Both knowledge of performance and knowledge of results were provided. |
| Timing of feedback | Both concurrent and terminal feedback were used. |
| Frequency of feedback | Faded feedback was provided. |
| Feedback information | Feedback included both error information and positive reinforcement. |

**Abbreviations:** UL-TOT = Upper-limb task-oriented training.
