# Supplementary material for: Upper‐Limb Paresis Severity Is Associated With Practice Dosage During Task‐Oriented Training in Subacute Stroke—An Exploratory Secondary Analysis
Source: Physiother Res Int. 2026 Aug 13;31(4):e70316. doi: 10.1002/pri.70316 (PMC13470900; doi:10.1002/pri.70316)
Supplement: Supplementary file 2 [file PRI-31-e70316-s002.docx]

**Figure S2: Normal Q - Q plot of standardized residuals from the final stepwise multiple linear regression model of practice dosage during UL-TOT.**

The standardized residuals were approximately aligned with the diagonal reference line, suggesting no major deviations from the assumption of normality.

**Abbreviations:** UL-TOT = Upper-limb task-oriented training.
