# Supplementary material for: Upper‐Limb Paresis Severity Is Associated With Practice Dosage During Task‐Oriented Training in Subacute Stroke—An Exploratory Secondary Analysis
Source: Physiother Res Int. 2026 Aug 13;31(4):e70316. doi: 10.1002/pri.70316 (PMC13470900; doi:10.1002/pri.70316)
Supplement: Supplementary file 3 [file PRI-31-e70316-s003.docx]

**Table S2: Demographics and clinical characteristics of participants (N=33).**

| **Participant** | **Age**  **(years)** | **Sex** | **Type of stroke** | **Lesion location** | **Lesion volume (cm^3^)** | **Poststroke duration (Days)** | **SAFE score** | **CSI score** | **PHQ-9 score** | **tDCS group** |
| --- | --- | --- | --- | --- | --- | --- | --- | --- | --- | --- |
| P1 | 45 | Male | Haemorrhagic | Subcortical | 39.5 | 154 | 1 | 8 | 10 | Sham |
| P2 | 73 | Female | Ischaemic | Cortical | 23.62 | 58 | 8 | 6 | 1 | Sham |
| P3 | 75 | Female | Ischaemic | Subcortical | NA | 69 | 4 | 7 | 9 | Active |
| P4 | 36 | Female | Ischaemic | Mixed | 76.95 | 60 | 3 | 6 | 7 | Sham |
| P5 | 58 | Male | Ischaemic | Subcortical | 2.81 | 32 | 2 | 9 | 6 | Sham |
| P6 | 48 | Male | Ischaemic | Subcortical | 14.69 | 39 | 1 | 4 | 16 | Active |
| P7 | 50 | Male | Ischaemic | Subcortical | NA | 68 | 5 | 4 | 2 | Active |
| P8 | 57 | Male | Ischaemic | Subcortical | 0.28 | 39 | 1 | 7 | 9 | Active |
| P9 | 52 | Female | Haemorrhagic | Subcortical | 14.99 | 25 | 4 | 7 | 17 | Sham |
| P10 | 65 | Female | Both | Mixed | 0.014 | 176 | 1 | 4 | 10 | Sham |
| P11 | 45 | Female | Ischaemic | Subcortical | 1.65 | 45 | 6 | 7 | 8 | Active |
| P12 | 26 | Male | Ischaemic | Mixed | NA | 145 | 1 | 10 | 5 | Active |
| P13 | 46 | Male | Ischaemic | Subcortical | 0.67 | 40 | 6 | 7 | 8 | Active |
| P14 | 38 | Male | Ischaemic | Mixed | 172.15 | 149 | 2 | 11 | 8 | Sham |
| P15 | 71 | Male | Ischaemic | Subcortical | NA | 42 | 7 | 4 | 2 | Active |
| P16 | 41 | Male | Ischaemic | Subcortical | 1.56 | 107 | 5 | 7 | 1 | Sham |
| P17 | 35 | Male | Haemorrhagic | Subcortical | 17.22 | 137 | 6 | 9 | 10 | Active |
| P18 | 65 | Male | Both | Subcortical | 22.37 | 174 | 1 | 7 | 5 | Sham |
| P19 | 61 | Female | Ischaemic | Subcortical | NA | 108 | 2 | 9 | 11 | Sham |
| P20 | 62 | Male | Both | Mixed | NA | 115 | 2 | 12 | 4 | Active |
| P21 | 72 | Female | Haemorrhagic | Mixed | 21.83 | 13 | 4 | 8 | 8 | Active |
| P22 | 49 | Male | Haemorrhagic | Subcortical | NA | 38 | 6 | 6 | 2 | Active |
| P23 | 49 | Male | Haemorrhagic | Subcortical | 25.18 | 157 | 1 | 9 | 14 | Active |
| P24 | 46 | Male | Haemorrhagic | Subcortical | 10.31 | 72 | 1 | 8 | 16 | Sham |
| P25 | 36 | Male | Ischaemic | Subcortical | 1.74 | 36 | 2 | 6 | 11 | Sham |
| P26 | 46 | Male | Haemorrhagic | Subcortical | 34.27 | 41 | 1 | 8 | 9 | Active |
| P27 | 71 | Female | Ischaemic | Subcortical | 3.73 | 43 | 1 | 8 | 10 | Active |
| P28 | 34 | Male | Haemorrhagic | Mixed | 79.16 | 68 | 1 | 7 | 1 | Sham |
| P29 | 44 | Male | Haemorrhagic | Subcortical | 15.88 | 34 | 6 | 5 | 16 | Active |
| P30 | 51 | Male | Haemorrhagic | Subcortical | 62.02 | 108 | 1 | 8 | 3 | Sham |
| P31 | 63 | Male | Ischaemic | Subcortical | NA | 55 | 5 | 8 | 3 | Active |
| P32 | 59 | Male | Ischaemic | Cortical | 0.16 | 15 | 1 | 2 | 6 | Sham |
| P33 | 56 | Female | Ischaemic | Cortical | NA | 50 | 7 | 6 | 1 | Sham |

**Abbreviations:** P: Participant; Both = Ischaemic and hemorrhagic; Mixed = Cortical and subcortical; SAFE = Shoulder Abduction and Finger Extension; CSI = Composite Spasticity Index; PHQ-9 = Patient Health Questionnaire-9; tDCS = Transcranial direct current stimulation; Active = Anodal tDCS; NA = Not available.

NA indicates missing data in the original dataset. Missing lesion volume values were handled using multiple imputation (50 imputations) in all statistical analyses.
