# Supplementary material for: Upper‐Limb Paresis Severity Is Associated With Practice Dosage During Task‐Oriented Training in Subacute Stroke—An Exploratory Secondary Analysis
Source: Physiother Res Int. 2026 Aug 13;31(4):e70316. doi: 10.1002/pri.70316 (PMC13470900; doi:10.1002/pri.70316)
Supplement: Supplementary file 5 [file PRI-31-e70316-s005.docx]

**Table S3:** **Practice dosage achieved during UL-TOT across participants (N = 33).**

| **Participant** | **Practice dosage**  **(Mean repetitions/session)** | **SD (repetitions)** | **Range**  **(repetitions)** |
| --- | --- | --- | --- |
| P1 | 56 | 14 | 25 - 80 |
| P2 | 188 | 25 | 130 - 240 |
| P3 | 96 | 25 | 55 - 145 |
| P4 | 152 | 35 | 54 - 195 |
| P5 | 75 | 14 | 55 - 103 |
| P6 | 70 | 17 | 31 - 106 |
| P7 | 200 | 46 | 113 - 257 |
| P8 | 97 | 20 | 57 - 130 |
| P9 | 230 | 83 | 38 - 360 |
| P10 | 50 | 6 | 39 - 62 |
| P11 | 156 | 22 | 120 - 205 |
| P12 | 67 | 22 | 31 - 96 |
| P13 | 263 | 49 | 178 - 339 |
| P14 | 64 | 12 | 49 - 94 |
| P15 | 162 | 46 | 105 - 230 |
| P16 | 175 | 18 | 137 - 204 |
| P17 | 209 | 31 | 153 - 253 |
| P18 | 77 | 8 | 60 - 88 |
| P19 | 91 | 8 | 77 - 109 |
| P20 | 43 | 5 | 34 - 53 |
| P21 | 306 | 124 | 115 - 445 |
| P22 | 277 | 59 | 142 - 346 |
| P23 | 57 | 5 | 49 - 65 |
| P24 | 102 | 19 | 63 - 126 |
| P25 | 168 | 42 | 94 - 239 |
| P26 | 67 | 10 | 45 - 82 |
| P27 | 41 | 5 | 30 - 46 |
| P28 | 41 | 10 | 30 - 63 |
| P29 | 279 | 63 | 130 - 392 |
| P30 | 47 | 8 | 30 - 58 |
| P31 | 161 | 39 | 98 - 237 |
| P32 | 90 | 34 | 39 - 150 |
| P33 | 357 | 104 | 171 - 498 |

Practice dosage: Mean number of repetitions per session across the 20 training sessions.

Participant 2 (P2) completed 19 of the 20 scheduled training sessions.

**Abbreviations:** SD = Standard deviation; UL-TOT = Upper-limb task-oriented training.
